# Evaluation of the PhunkyFoods intervention on food literacy and cooking skills of children aged 7–9 years: a cluster randomised controlled trial in Yorkshire Primary Schools UK

**DOI:** 10.1186/s13063-022-06558-5

**Published:** 2022-08-01

**Authors:** Karen L. Vaughan, Janet E. Cade, Marion M. Hetherington, Jennie E. Cockroft, Mirjam M. Heinen, Holly Rippin, Charlotte E. L. Evans

**Affiliations:** 1 University of Leeds, Leeds, UK; 2 Purely Nutrition Ltd., Marlow, UK; 3 WHO European Office for Prevention and Control of Noncommunicable Diseases (NCD Office), Copenhagen, Denmark

**Keywords:** Cluster RCT, Food literacy, Cooking skills, Complex intervention, Childhood obesity prevention, Primary schools, Protocol

## Abstract

**Background:**

Childhood obesity rates more than double during primary school in England. Acquiring competent cooking skills is a key part of children’s education that can lead to improved knowledge of a healthy lifestyle and dietary behaviours. Evaluation of the impact of ‘PhunkyFoods’, a school-based food and nutrition education programme, will assess food literacy, cooking skills and dietary behaviour in primary-school children.

**Methods:**

A cluster randomised controlled trial will be undertaken in 28 primary schools in North Yorkshire, UK, including a total population of children aged 7–9 years (*n* = 420). The trial has two arms: (a) the intervention group receiving PhunkyFoods programme (*n* = 210) and (b) the wait-list control group receiving the usual school curriculum (*n* = 210). The intervention ‘PhunkyFoods’ will be delivered by Purely Nutrition Ltd. The participating school staff are supported with training, policy development and access to resources to improve the delivery of nutrition education. Children participate through whole school assemblies, classroom activities, and after-school clubs about food preparation, cooking healthy meals and healthy living. Schools, parents and children have access to healthy meal recipes through the PhunkyFoods website. The primary outcomes are differences in food literacy and cooking skills scores between control and intervention arms after 12 months of the intervention and adjusted for baseline values. The secondary outcome is differences in fruit and vegetable intake between the arms after 12 months (adjusted for baseline). Treatment effects will be examined using mixed ANOVA and regression analysis. Primary analyses will adjust for baseline food literacy and cooking skills scores and secondary analysis will adjust for pre-specified baseline school and child level covariates.

**Discussion:**

The PhunkyFoods programme is a flexible menu of options for schools to choose from, making this a highly complex intervention. Following Medical Research Council guidance, research perspectives will focus on effectiveness and theory-based approaches: to what extent the intervention produces the intended outcomes in real-world settings and what works in which circumstances.

**Trial registration:**

ISRCTN ISRCTN68114155. Prospectively registered on 22 October 2021

**Supplementary Information:**

The online version contains supplementary material available at 10.1186/s13063-022-06558-5.

## Administrative information

Note: The numbers in curly brackets in this protocol refer to SPIRIT checklist item numbers. The order of the items has been modified to group similar items (see https://uk01.l.antigena.com/l/No6NZkdmCCqK54rxvBrmWWdkRMJMZezCpgvwMZggQBsXfTE5JxwRkvfLlKx0U70lwk7cMxP4G-JXA9DRXoSHskRP35mvo0zLdHo13fmjz3NUC2JsGZx7J2EDnMtOmO38H5AIbd3MRNSKY9lc58ocvVAIu9e8SKQUoh9njrlxFgTyLFNTJYXVHyGkSTxrbtVR3WJgqPGboeXzZpCi3bHGJTQ6-boK).

**Table:** 

Title {1}	Evaluation of the PhunkyFoods intervention on food literacy and cooking skills of children aged 7–9 years: a cluster randomised controlled trial in Yorkshire Primary Schools UK Cooking In Yorkshire / PhunkyFoods Cluster-RCT
Trial registration {2a and 2b}.	ISRCTN68114155
Protocol version {3}	Issue date: 14th January 2022 Protocol number: 01 Author: KV, JaC, MaH, JeC, HR, MiH, CE
Funding {4}	The PhunkyFoods programme in North Yorkshire is funded by Nestlé For Healthier Kids and delivered by Purely Nutrition Ltd. The lead researcher is supported by the University of Leeds as part of a PhD project. This study has been produced with financial assistance within the context of the WHO European Office for the prevention and control of Prevention and Control of Non-communicable Diseases.
Author details {5a}	Karen Vaughan MBA, MSc (KV) Food Science and Nutrition, University of Leeds, Leeds, UK. Professor Janet Cade (JaC) Food Science and Nutrition, University of Leeds, Leeds, UK. Professor Marion Hetherington (MaH) Psychology, University of Leeds, Leeds, UK. Dr Jennie Cockroft (JeC) Director of Purely Nutrition Ltd. Dr Holly Rippin (HR) WHO European Office for Prevention and Control of Noncommunicable Diseases (NCD Office) Dr Mirjam Heinen (MiH) WHO European Office for Prevention and Control of Noncommunicable Diseases (NCD Office) Dr Charlotte Evans (CE) Food Science and Nutrition, University of Leeds, Leeds, UK
Name and contact information for the trial sponsor {5b}	c/o Prof. Jason Halford G.02 Psychology Building University of Leeds Leeds LS2 9JT England, United Kingdom +44 (0)113 343 6678 J.Halford@leeds.ac.uk
Role of sponsor {5c}	The Purely Nutrition staff will have some role in the data collection activities across the 28 school sites, working with the University of Leeds research team staff. Nestlé Healthy Kids and Purely Nutrition will not have any role in the study design, management, analysis and interpretation of the data; writing of the report; or decision to submit the report for publication. The ultimate authority over all the research activities resides with the University of Leeds.

## Introduction

### Background and rationale {6a}

#### Childhood obesity in the UK

Childhood obesity rates typically double during primary school in England and prevalence at age 4–5 years in reception classes has increased to 14% in 2020/2021 [1]. Good nutrition and maintaining a healthy weight in childhood help to prevent obesity and diet-related ill health later in life [2]. Knowledge about nutrition and cooking healthy meals helps people to live healthier lives [3–5].

#### School-based nutrition programmes: existing knowledge

A systematic literature review of successful primary school-based nutrition programmes has highlighted the value of experiential curriculum strategies to develop food literacy and cooking skills in children. Programmes are more likely to be successful if they have multiple strategies, parental involvement and focus specifically on vegetable intake [6].

The PhunkyFoods programme aims to help early years settings and primary schools to deliver a whole-settings approach to healthy lifestyles and to engage with all pupils, and their families, in promoting tangible health behaviour change in a fun, lively and positive manner. The cooking skills elements of the PhunkyFoods programme delivered in schools are supplemented with additional components such as online videos demonstrating knife and other cooking skills [7].

The feasibility study undertaken in 2019 by Sahota and colleagues [8] identified key outcomes of the programme but was not powered to detect definitive effectiveness. However, results did show the potential of the programme to increase knowledge of healthy lifestyle and dietary behaviours.

This research will build on the previous feasibility trial by implementing recommendations for enrolment and baseline data collection with a fully powered phase 3 trial design. The trial will investigate the impact of the PhunkyFoods programme on dietary behaviour, nutrition knowledge and cooking skills for primary-aged children aged 7–9 years. This age group was chosen as relevant for study as this population is also targeted by the World Health Organization Child Obesity Surveillance Initiative (COSI) as an important area for research. This UK trial will provide supplementary contextual data to complement the current COSI survey 2021–2023, which includes new questions about children’s food preparation and cooking skills in this age group [9].

### Objectives {7}

The main aim is to assess the impact of the PhunkyFoods healthy lifestyle intervention programme, (developed and refined by Purely Nutrition), on food literacy, cooking skills and fruit and vegetable intake of children using usual practice in primary schools as the comparator. Intervention effects will be examined at 12 months. The cost-effectiveness of the intervention will be assessed from a societal perspective. In addition, differences in outcomes will be explored by model fidelity in each school setting, other healthy living initiatives in the local area and socioeconomic status using percentage eligibility for free school meals (%FSM) and the index of multiple deprivation (IMD) as covariables.

The PhunkyFoods programme is a flexible menu of options for schools to select from, making this a highly complex intervention. Following the Medical Research Council guidance on the evaluation of complex interventions, research perspectives will focus on effectiveness and theory-based approaches: the overall objective of the study is to explore to what extent the intervention produces the intended outcomes in real-world settings and what works in which circumstances [10].

The research hypotheses are as follows:

1. The PhunkyFoods intervention group will show higher food literacy and cooking skills than the control group measured by mean scores.
2. The PhunkyFoods intervention group will show a higher intake of fruit and vegetables than the control group measured by mean scores.
3. Schools in the intervention arm that choose more ‘active ingredients’ (intervention components) from the flexible menu of options will have better outcomes than those schools that choose less ‘active ingredients’.
4. The covariates, %FSM and IMD, will have a mediating impact on the outcomes for schools in the intervention arm.

### Trial design {8}

The PhunkyFoods evaluation design is a parallel, cluster randomised controlled trial, with two arms and with a 1:1 allocation ratio. The intervention arm will receive the PhunkyFoods programme starting in May 2022, and the wait-list control arm will receive the PhunkyFoods programme after the final research data collection, with training from May 2023 (superiority trial). If the trial is not able to recruit 28 schools within the recruitment period, then the research team will consider an unequal allocation ratio with a smaller number of schools in the control group or widening the geographical recruitment area. The impact of this is discussed in the section on sample size.

## Methods: participants, interventions and outcomes

### Study setting {9}

All state mainstream primary schools in Harrogate (*n* = 74) and Selby (*n* = 40) areas of North Yorkshire are eligible for inclusion and will be invited to express an interest in the study. Schools will be approached until the required sample size of (*n* = 28) schools is achieved.

### Eligibility criteria {10}

The following are the exclusion criteria: schools with fewer than 20 pupils in the relevant year groups (year 3 and/or year 4); schools that are unable to commit to the two data collection points in March 2022 and March 2023 for the identified class of children will also be excluded from the study.

### Who will provide informed consent? {26a}

Informed consent will be requested at three stages:

1. School consent: Headteachers will be provided with an information sheet and consent form for the Study. Details of the PhunkyFoods Programme and the research study will be explained via a 30-min School Briefing session on Microsoft Teams by the lead researcher (KV). If they decide to participate and the school meets the eligibility criteria, informed consent for the school to participate will be signed by the headteacher and a class identified to participate in the study.
2. Parent consent: Once the school consent has been obtained, a parent information sheet will be sent out for all the parents/carers for children in the identified class with an opt-out clause. If parents do not want their child to participate in the research study, they can either email or ring the school. This information will then be passed to the research team to update the recruitment flow diagram. Parents will be given 2 weeks if they wish to withdraw their child from the research study. This will mean that all children will still participate, and no child will be excluded from the PhunkyFoods programme activities. This includes the data collection activities; however, their data will not be used for the purposes of research if the parent opted out.
3. Child assent: The two data collection surveys for children will have a consent tick box on the front page. The lead researcher will explain that no individual child will be identified from the study, and the information will not be used beyond the research team. Children who are happy with the surveys to be used in the research study will be asked to tick the consent box on the front page of the surveys. At the beginning of the research activity, the researcher will explain to the children about the work of the University on Food Science and Nutrition and ask them to complete the surveys as part of the study.

### Additional consent provisions for collection and use of participant data and biological specimens {26b}

N/A—no biological specimens are collected as part of this trial.

### Interventions

#### Explanation for the choice of comparators {6b}

The control group schools will receive their usual existing school curriculum for food science as delivered in the Food Technology schemes of work, without any support from the PhunkyFoods Programme. This may include specific nutritional components, designed and delivered by the school. In the UK, the Department for Public Health sets out the topics that should be covered in Food Technology lessons for primary schools [11] but in reality, this is likely to look different across school settings. We will ensure we collect relevant information on what schools are currently doing in relation to food knowledge and skills and include this information in the analysis as an additional independent variable if deemed appropriate.

#### Intervention description {11a}

The PhunkyFoods Programme is a flexible menu of 8 ‘active ingredients’, which schools can select from. The Education Endowment Foundation (EEF) uses the term ‘active ingredients’ to describe intervention components in a school intervention [12]. The PhunkyFoods Logic Model in Additional file 1 shows the behaviour theory for the PhunkyFoods programme and implementation planning.

The PhunkyFoods programme has a detailed delivery manual with implementation guidance and resources for Engagement & Development Coordinators (EDCs) to deliver each of the 8 active ingredients in partnership with schools [13]:

- Active ingredient 1: Whole setting staff training in the PhunkyFoods Programme with additional Continuous Professional Development (CPD) opportunities available for schools to opt into. Examples of CPD opportunities include National Level 2 Award in Nutrition and Health of School Aged Children, Food Prep in the Classroom and Setting Up and Running a Cook Club.
- Active ingredient 2: Whole Setting Audit (Health Check) and action planning support around the Whole School Approach to Health. This consists of an initial meeting with a member of the Senior Leadership Team (SLT) to work through the Health Check document, then co-production of an action plan for development priorities agreed.
- Active ingredient 3: Policy evaluation and updating of Whole School Food and Packed Lunch policies.
- Active ingredient 4: Whole school activities such as assemblies and pupil workshops on a range of topics relating to Phunky FOOD. Examples include Eatwell, Strive for 5, Drain your Drinks, Bag-A-Breakfast, Top Teeth, A Healthy Lunch, Snack Attack, Food Waste, Phunky FIT (Get Active) and Phunky MINDS (Resilience, Feelings, Relationships, Anti-Bullying).
- Active ingredient 5: Experiential curriculum/classroom-based activities. Examples include Design and Technology Scheme of Work; Personal Health and Social Education Scheme of Work; Topic Based Activities; Planning, Preparation and Assessment solutions (healthy eating and physical activity); Phunky15 (physical activity); and Mindful Moments.
- Active ingredient 6: Extra-curricular activities. Examples include Breakfast Club, Cookery Club, Gardening Club and After-schools Club.
- Active ingredient 7: Student-led activities. The Phunky AMBASSADORS programme involves mentoring students to deliver key healthy lifestyle messages through peer-to-peer learning.
- Active ingredient 8: Parent engagement activities. Examples include Parent/Child Cook Clubs, Parent Workshops, Parent Stay and Play Sessions, Parent Health Promotion Events and Parent Communication Material (newsletter text; email snippets; display assets).

#### Criteria for discontinuing or modifying allocated interventions {11b}

Participating schools in the intervention arm will be given the freedom to choose from the flexible menu of active ingredients in the programme and work at the pace that is right for the individual school setting. In line with the PhunkyFoods Logic Model (Additional file 1), schools will be encouraged to participate in active ingredients 1 and 2 within the first 3 months as part of the model fidelity. Whilst the programme pace is led by schools, the EDCs will support and encourage participating schools to consider active ingredient 3 by 6 months of the programme and will highlight opportunities to select ‘Active Ingredients’ 4–8 after 6 months. This flexibility is indicative of the complexity of the trial and hence the theory-based approach to research perspectives and hypotheses which aim to understand the interplay of mechanisms and context in evaluating complex interventions [10].

#### Strategies to improve adherence to interventions {11c}

The PhunkyFoods programme is run by Purely Nutrition. All EDCs are employed by Purely Nutrition and supported with training and guidance on how to implement the programme according to the Programme Manual [13] and the Logic Model, whilst providing the flexibility for schools to work at the pace that is right for their setting. The study will collect data on of how many components each school delivers and this will be included as part of the analysis.

#### Relevant concomitant care permitted or prohibited during the trial {11d}

N/A—no concomitant care is permitted or prohibited as part of this trial.

#### Provisions for post-trial care {30}

N/A—no provisions for ancillary and post-trial care as part of this trial.

### Outcomes {12}

At the time of trial registration, it was thought that the outcome measures would be measured at baseline (T1) and then at 8 months post-intervention delivery (T2), identifying September as the start time of the intervention. However, in recognition that the training phase of the programme delivered in May to July includes active ingredients 1 and 2, it is felt more appropriate to include this phase in the time period from baseline to the final value. Therefore, the outcomes will be measured at baseline (T1) and then 12 months (T2) after the intervention starts, including the initial training for teachers.

#### Primary outcome measures

The primary outcome measures are as follows:

1. Food literacy will be measured by the Tool for Food Literacy Assessment in Children (TFLAC - UK) at baseline (T1) and at 12 months after intervention delivery (T2). The Food Literacy outcome is composite and contains numerical values for food systems knowledge, cooking skills, cooking knowledge, nutrition knowledge and self-efficacy. The original questionnaire developed by Amin et al. [14] Tool for Food Literacy Assessment in Children (TFLAC) was developed by a panel of food and nutrition experts in three phases for content validity, with test-retest reliability and internal consistency assessed amongst children (*n* = 706) aged 9–11 years. Food literacy domain-specific Cronbach alpha values were food systems knowledge (0.80), cooking skills (0.94), cooking knowledge (0.63), nutrition knowledge (0.83) and self-efficacy regarding eating (0.98), and intraclass correlation coefficients were 0.64−0.70 (*P* < 0.001).
  The TFLAC was adapted for use in the UK by consulting with EDCs at PhunkyFoods during an away day on 10 September 2021. Terminology was changed from US English words to equivalent UK English words and then further checks on the survey language were undertaken by the authors of this protocol. Example changes included ‘measuring cup’ to ‘measuring jug’ and ‘oven mitt’ to ‘oven glove’. No other changes were made to the content, structure, order, or phrasing of questions in the survey design that could have an impact on internal consistency.
2. Cooking skills will be measured by the CooC11 child survey by Dean et al. [15] at baseline (T1) and at 12 months after intervention delivery (T2). CooC11 and CooC7 are two measures of cooking competence developed and reviewed by an expert panel, based on new recommendations about children’s developmental skills and are relevant for this specific age group [15, 16]. The internal consistency reliability of CooC11 was good, with a Cronbach score of 0.86 and a temporal stability rating ICC of 0.91. The measure was shown to be responsive to change in the cooking camp intervention with differences in pre-cooking camp mean (SD) of 21.75 (7.89) to post camp mean of 26.13 (8.89) [15].

#### Secondary outcome measures

The secondary outcome measures are as follows:

1. Total fruit and vegetable intake will be measured using a shortened version of the Child Assessment of Diet Evaluation Tool (CADET) [17] at baseline (T1) and at 12 months after intervention delivery (T2). The CADET is a 24-h food diary that measures the nutritional intake of children and has been validated for use with children aged 3–11 years [17, 18]. A sample of children (*n* = 67) completed the diary with a mean age of 9.3 years in the validation study [17]. The proposed CADET fruit and veg survey uses a sub-section of CADET, which assesses dietary information on fruit and vegetables only. This shortened version was chosen by the authors to make it easier for parents to complete and for the potential of a shorter version to increase the survey return rate.

#### Participant timeline {13}

**Fig. 1:**
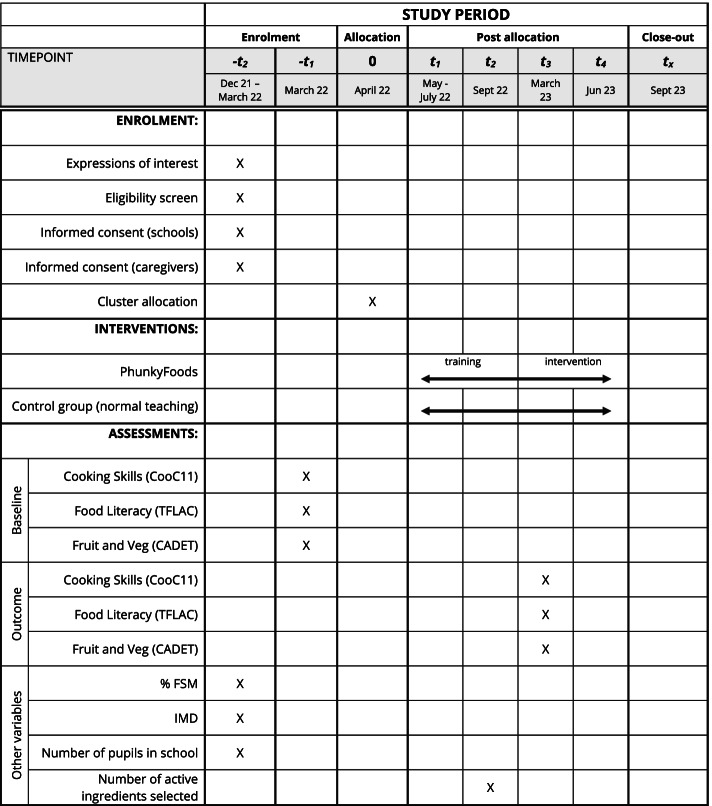
Schedule of enrolment, intervention and assessments

Participant timeline is presented in Fig. 1.

#### Sample size {14}

The study by Dean and colleagues in 2019, based on 469 participants and using CooC11 to assess cooking competence, showed a significant increase (*p* < 0.01) in cooking competence at follow-up after the camp-based intervention compared with results at baseline [15]. The results of CooC11 at baseline (mean (SD)) were 21.75 (7.89) and at follow-up 26.13 (8.89). The difference in cooking competence scores was therefore approximately 5 units, the equivalent of a moderate effect size (Cohen’s *d* = 0.52).

A power calculation was undertaken using STATA based on this moderate effect size of the Dean study. To detect this effect size with a minimum of 90% power and using a 2-level model with individuals clustered within schools (individual schools are being recruited rather than individual children), 13 clusters are needed in each arm. This calculation was based on the assumption that a mean of 15 children would be included in each school, and therefore, 195 children would be needed in each arm. The power is reduced when a large proportion of variation is at the school level (most of the variation will be at the individual level); in other words, children within a school may be more similar to each other than children at another school and therefore a conservative estimate of 20% of variation at the school level was assumed. It is likely that it is less than this, but there is too little information available related to cooking competence to be able to estimate it with more certainty. Due to the reasonable possibility that schools may drop out of the study, we aim to recruit 14 schools per arm (28 schools in total).

#### Recruitment {15}

Eligible schools will be recruited through email, social media, newsletters and follow-up telephone calls. Networks such as North Yorkshire County Council, Huntington Research School and Red Kite Teaching School Alliance will be used to help promote the project in newsletters and social media. Those schools that express an interest in the project will be sent a ‘Cooking in Yorkshire’ Poster, a Headteacher Information Sheet with details about the study, aims and methodology, and invited to a 30-min online school briefing to discuss the project and research design.

**Fig. 2:**
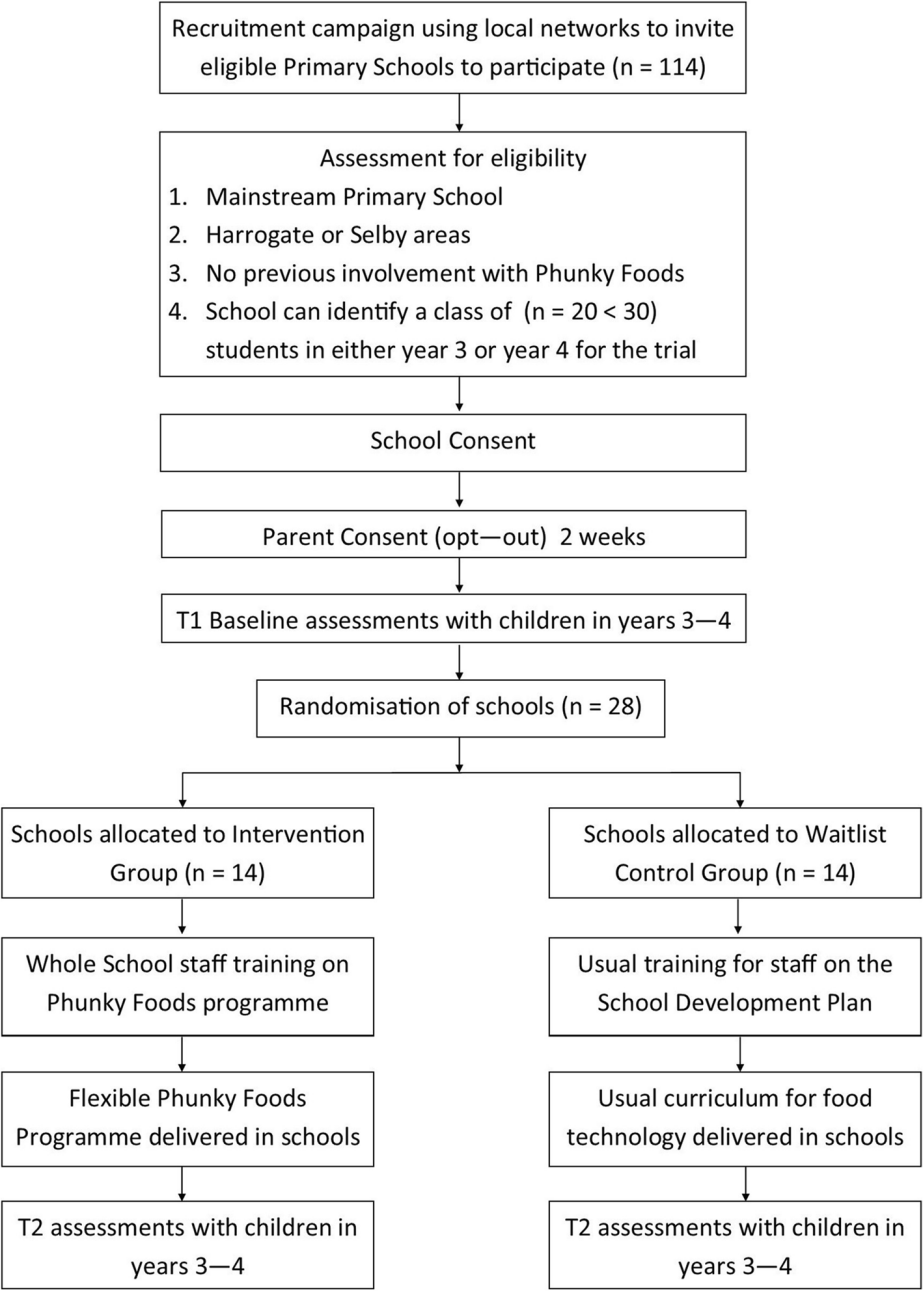
Flowchart of the trial procedures. Children who are in years 3–4 at UK primary schools in Yorkshire are aged 7–9 years

Once schools that have received a briefing about the project understand the randomised controlled trial design and data collection elements of the study, they will be asked to sign the Headteacher Consent Form and allocate one class for the research project. Parental and child consent procedures will then take place and have already been outlined early. A flowchart for the trial procedure is shown in Fig. 2.

### Assignment of interventions: allocation

#### Sequence generation {16a}

A computer-generated randomisation sequence will be used to allocate schools into the intervention and wait-list control arms of the trial. Block randomisation will be used to ensure equivalence between intervention and waitlist-control schools. Furthermore, schools will be stratified according to region (e.g. Selby or Harrogate) and whether they are above or below the England median for free school meals eligibility [19]. The randomisation will be conducted immediately after T1 baseline data is collected.

#### Concealment mechanism {16b}

A computer-generated randomisation sequence will be used to allocate schools into the intervention and wait-list control arms of the trial. The research team will not be fully blinded to the group allocation. Schools will be informed of which group they have been allocated to and therefore the schools will not be blind to their allocation.

#### Implementation {16c}

The lead researcher (KV) will enrol participants in the study and allocate them a unique ID number. One member of the research team (CE), blinded to the schools, will generate the allocation sequence using the school ID numbers but not the school names. The lead researcher (KV) will then inform schools which group they have been allocated to.

### Assignment of interventions: blinding

#### Who will be blinded {17a}

There will be full blinding at T1 baseline data collection by trial participants, schools and the research team, since randomisation will take place after data is collected. This is a pragmatic trial evaluating the effectiveness of a food skills-based intervention and therefore it is not possible for trial participants, schools or parents to be blinded after assignment to the intervention groups. We plan for the blinding of outcome assessors at T2 by using additional research team members from the University of Leeds to undertake data collection in the participating schools. This will be dependent on access to further funding being sought. It will not be possible for the data analyst to be blinded, since this work is in support of a PhD thesis by the main author of the study.

#### Procedure for unblinding if needed {17b}

N/A—no procedure for unblinding as part of this trial as participants are not blinded to the allocated group.

### Data collection and management

#### Plans for assessment and collection of outcomes {18a}

Data collection will be via paper surveys at two time points: baseline (T1) and 12 months post-intervention (T2). The two child surveys on Food Literacy and Cooking Skills will be conducted on the school premises in a whole class setting. The fruit and veg diet diary (CADET) will be sent home to parents in paper format, asking them to complete it and send it back to the school.

#### Plans to promote participant retention and complete follow-up {18b}

Short articles about the research project will be sent out in School Newsletters in the half-term before data collection with a photograph of the main researcher (KV) to make the message more personal. It is hoped that this personal approach will encourage the completion of the CADET fruit and veg survey by parents.

#### Data management {19}

All paper-based data will be collected by the lead researcher and held in locked filing cabinets in a locked office. There will need to be some training undertaken for the coding work on diaries and questionnaires to ensure a consistent approach across the research team. For data entry, a 5% sample will be checked by another member of the research team to monitor error rates. The data will be inputted into MS EXCEL by the lead researcher (KV), exported to SPSS and STATA for analysis and saved on the University of Leeds SAN (Storage Area Network), which comprises enterprise-level disk storage and file servers located in physically secure data centres with appropriate fire suppression equipment. The electronic data will be accessible only to the research team.

#### Confidentiality {27}

Individual participants will be given an anonymous unique identity number. All participating schools and families will be informed that the data provided will be treated confidentially. It will be explained that the research team are the only people who will look at the data and that the published reports of results will not identify any individuals either before, during or after the trial.

#### Plans for collection, laboratory evaluation and storage of biological specimens for genetic or molecular analysis in this trial/future use {33}

N/A—no biological specimens are collected as part of this trial.

## Statistical methods

### Statistical methods for primary and secondary outcomes {20a}

Baseline demographic characteristics for the participating schools will be summarised using means and 95% confidence intervals for continuous variables and frequencies and percentages for categorical variables. Contextual data such as %FSM eligibility, IMD and Public Health England data from National Child Measurement Programme (NCMP) [1] will be provided at school and Lower Super Output Area (LSOA) [20] levels respectively in a summary table.

Primary analysis of the effectiveness of the intervention on outcome data for the trial arms will be compared using two-way mixed analysis of variance (ANOVA) and multivariable regression models. The statistical analyses will be conducted using the following: food literacy score, cooking skills score and fruit and vegetable intake score as the dependent variables; time (T1 and T2) as the repeated measures independent variable; and treatment (intervention/control) as the independent groups variable. Effect size coefficients will be reported with measures of variation as well as Cohen’s *d* to determine the relative size of the effectiveness. We have identified the key outcomes on which we are basing our evaluation and any additional results will be interpreted cautiously and with the awareness that multiple testing is an issue. To reduce this risk, we will consider using statistical significance at the 1% level.

### Interim analyses {21b}

N/A—no interim analyses are planned as part of this trial.

### Methods for additional analyses (e.g. subgroup analyses) {20b}

Secondary analysis will use a theory-based approach to explore the interplay of mechanisms and context [10], including model fidelity. This will involve adjusting for pre-specified baseline school and child level covariates to investigate the impact of the intervention arm for individual clusters. Multiple regression will be used with the number of intervention components as a predictor variable. In addition, %FSM and IMD will be used in a linear model as predictor variables. The study will have low power to detect all but the largest differences in subgroups.

### Methods in analysis to handle protocol non-adherence and any statistical methods to handle missing data {20c}

Missing data will be reported and associations between outcomes considered. A sensitivity analysis will be undertaken if deemed appropriate, depending on the extent of the missing data.

### Plans to give access to the full protocol, participant-level data and statistical code {31c}

As stated in the information sheets for headteachers and parents, no individual participant-level data will be available outside of the research team. The datasets analysed during the current study and statistical code are available from the corresponding author on reasonable request, as is the full protocol.

### Oversight and monitoring

#### Composition of the coordinating centre and trial steering committee {5d}

Once recruitment is underway, a Trial Steering Group will be set up comprising representation from the University of Leeds, Purely Nutrition Ltd., North Yorkshire County Council and Primary Schools in Harrogate or Selby. The Trial Steering Group will meet online, at a minimum, 4 times during the trial: an initial meeting to discuss the role of the group, 2 weeks before both data collection time points and at the end of the Trial for a close-out endpoint discussion and final feedback.

The coordinating centre Research Team at the University of Leeds will provide oversight of the whole trial design, day-to-day management of the trial, data management processes, analysis, report writing and decisions on publications. The coordinating centre research team will meet every 8 weeks throughout the trial to monitor the progress and data collection, consider risks and address any issues raised by the Trial Steering Group members.

#### Composition of the data monitoring committee, its role and reporting structure {21a}

The coordinating centre research team (KV, CE, JeC and MaH) will provide the function of data monitoring, including the oversight of the data management processes and quality control. This work will take place as part of the formal PhD supervision processes at the University of Leeds, the sponsor of the study. The lead researcher (KV) will report to the Trial Steering Group on data monitoring issues on behalf of the coordinating centre research team.

#### Adverse event reporting and harms {22}

Adverse events (for example, any minor mishaps or injuries that occur due to food preparation or food cooking activities) will be identified in discussion with Purely Nutrition Ltd., recorded in a secure place and reported in a timely fashion to the Steering Group.

#### Frequency and plans for auditing trial conduct {23}

The principal researcher (KV) from the research team will undertake 4 audit visits to schools in the intervention arm during the delivery phase to observe the activities, consider trial conduct and consider model fidelity issues. This will not be independent from the sponsoring organisation. Schools will be chosen at random from the participating cluster sites and then dates arranged by liaison with the PhunkyFoods representative on the Trial Steering Group.

#### Plans for communicating important protocol amendments to relevant parties (e.g. trial participants, ethical committees) {25}

Any important protocol amendments will be decided by the Coordinating Centre Research Team and communicated to relevant stakeholders, for example, the University Ethics Committee, Trial Steering Group, trial participants, the trial registry (ISRCTN) and journals.

#### Dissemination plans {31a}

The research team intends to disseminate the trial results through publication in an open-access relevant academic journal. There is no intention to share the data with anyone outside the university at this stage although any requests will be considered by the research team.

## Discussion

The World Health Organization (WHO) Regional Office for Europe Data Dashboard shows that obesity continues to rise at an alarming rate [21]. The global target of halting the rise of obesity is going in the wrong direction in the WHO European Region and so effective interventions to improve diet quality are needed. The overall findings from the fourth round of the WHO COSI report showed that almost 1 in 3 children aged 6–9 are living with overweight or obesity. The highest prevalence of childhood overweight and obesity were observed in Cyprus, Spain, Greece and Italy, where over 40% of boys aged 6–9 years and over 30% of girls aged 6–9 years were living with overweight (including obesity) [22]. The PhunkyFoods intervention could be a UK case study example that could inform similar interventions in other Member States.

Given the complex nature of the intervention and the contextual factors, it will not be possible to examine the efficacy of the PhunkyFoods programme as an experimental setting. Instead, the research perspective focuses on effectiveness and theory-based approaches. The cluster randomised controlled trial design and plans for secondary analysis will allow for some investigation and discussion into what works in what circumstances and why.

The additional level of complexity for this study is the flexibility in which schools choose from the active ingredients in the Logic Model. Whilst this reflects the real-world context of the study, it also limits the statistical power for cluster comparison between settings since the intervention will likely look very different in each school. It is hoped that the secondary analysis using complex regression, using the number of active ingredients, %FSM and %IMD as predictor variables, combined with observations from the audit visits, will enable some discussion about the effectiveness of particular settings.

## Trial status

Protocol version 1, 14 January 2022. Recruitment of participants began 01/12/2021 and will end on 31/3/2022.

## Supplementary Information

**Additional file 1.** Logic model - PhunkyFoods Intervention in primary and early years settings.

**Additional file 2.** Consent Materials.

## Data Availability

The final trial data for this protocol can be supplied on request to the research team at the University of Leeds. The research materials (information sheets, consent forms, surveys) will be available upon request to KV by emailing a request to mc17kv@leeds.ac.uk.

## Abbreviations

ANOVA: Analysis of variance
CADET: Child Assessment Diet Evaluation Tool
COSI: Child Obesity Surveillance Initiative
EDCs: Education and Development Coordinators
EEF: Education Endowment Foundation
FSM: Free school meals
IMD: Index of multiple deprivation
LSOA: Lower super output area (a geographic hierarchy designed to improve the reporting of small area statistics in England and Wales)
NCMP: National Child Measurement Programme
TFLAC: Tool for Food Literacy Assessment in Children
WHO: World Health Organization
