# Supplementary material for: Evaluation of the PhunkyFoods intervention on food literacy and cooking skills of children aged 7–9 years: a cluster randomised controlled trial in Yorkshire Primary Schools UK
Source: Trials. 2022 Aug 1;23:618. doi: 10.1186/s13063-022-06558-5 (PMC9344772; doi:10.1186/s13063-022-06558-5)
Supplement: Supplementary file 1 — Additional file 1. Logic model - PhunkyFoods Intervention in primary and early years settings. [file 13063_2022_6558_MOESM1_ESM.pdf]

Appendix 1: Logic model - PhunkyFoods Intervention in primary and early years settings

Education Endowment Foundation

Problem (why?) →

School food environment

- School meals and packed lunches do not meet nutrition guidelines.
- School Food Policy does not promote a whole school approach to health.

Teachers

Teachers lack access to training, resources and confidence in teaching a relevant curriculum for food literacy and cooking skills.

Students

- Public Health data (NCMP) shows that obesity levels double from Y1 to Y6 in Primary Schools[1].
- Students lack food literacy, cooking skills, which leads to poor diet choices[2].
- Diet choice behaviours contribute to poorer health outcomes.

Parents

- Obesity and overweight in the UK is increasing, particularly in families in areas of high deprivation.
- The dialogue between parents and schools in relation to health can be problematic and could be supported with facilitation.
- Some parents may not have access to recipes and confidence to cook nutritious meals at home.
- Family dietary attitudes, behaviours and priorities may need to change in order to improve health outcomes.

Intervention Description (what?) →

Active ingredient 1: Whole setting staff training in the PhunkyFoods Programme[3], plus additional CPD opportunities available e.g., National Level 2 Award in Nutrition and Health of School Aged Children | Food Prep in the Classroom | Setting Up and Running a Cook Club.

Active ingredient 2: Whole Setting Audit (Health Check) and action planning support around the Whole School Approach to Health.

Active ingredient 3: Policy Setting/Updating e.g., Whole School Food Policy | Packed Lunch Policy.

Active ingredient 4: Whole School Activities e.g., assemblies, pupil workshops on a range of topics relating to Phunky FOOD (Eatwell guide, Strive for 5, Drain your Drinks, Bag-A-Breakfast, Top Teeth, A Healthy Lunch, Snack Attack, Food Waste), Phunky FIT (Get Active) and Phunky MINDS (Resilience, Feelings, Relationships, Anti-Bullying).

Active ingredient 5: Experiential curriculum or Classroom-based Activities[4] e.g., D&T Scheme of Work | PSHE Scheme of Work | Topic Based Activities | PPA-solution (healthy eating and physical activity) | Phunky15 (physical activity) | Mindful Moments.

Active ingredient 6: Extra-curricular Activities e.g., Breakfast Club | Cookery Club | Gardening Club | After-schools Club.

Active ingredient 7: Student-Led Activities e.g., Phunky AMBASSADORS (delivering key healthy lifestyle messages through peer-to-peer learning.

Active ingredient 8: Parent Engagement Activities[5] e.g., Parent/Child Cook Clubs | Parent Workshops | Parent Stay and Play Sessions | Parent Health Promotion Events | Parent Communication Material (newsletter text; email snippets; display assets).

Implementation Activities (how?) →

Training

- Whole School staff training on the PhunkyFoods Programme.
- Individual teaching staff training to support CPD awards and accreditation.
- Skills training on food preparation delivery.
- Cooking skills and healthy eating workshops and training for parents.

Coaching

- Modelling delivery of whole school activities and workshops.
- Coaching support for SLT and GB to update school food policy.
- Coaching for individual teachers for co-delivery of the classroom-based activities.
- Coaching support for pupils - Phunky AMBASSADORS.

Monitoring

- Records of visits to schools.
- Action Plan monitoring through health checks.
- Activity record of parental engagement.
- Annual Schools Survey report.
- Evaluate all training and workshops.

Education materials

- Resources e.g., giant floor playmats (Eatwell Guide | Eat A Rainbow), food models, flashcards, storybooks with healthy eating themes, music/songs with healthy eating themes, activity workbooks (Social History of Food; Religion and Food).
- Online portal access to curriculum and extra curricula resources to support active ingredients.
- Online website access to recipes.

Implementation Outcomes (how well?) →

Short term (3 months)

Fidelity:

- Staff demonstrate willingness to engage in the Phunky Foods programme through active ingredients 1 – 2.

Acceptability:

- School completed a Whole Setting Audit (health check).
- Whole school (or key stage) teaching staff have undertaken the training.

Reach:

- Whole school commitment to use PhunkyFoods curriculum and resources.

Medium term (6 – 18 months)

Fidelity:

- Staff show understanding of how to use the curriculum materials and can deliver healthy eating and physical activities to children using the resources.
- Embedded use of schemes of work in the curriculum (1 – 2 years).
- SLT agree to review and update the School Food Policy (active ingredient 3).
- School selects from optional active ingredients 4 – 8 for delivery.
- SLT identify staff for additional coaching support where appropriate.

Acceptability

- Annual schools' surveys show the teachers are responding to the programme.
- Pupil surveys show high acceptability of the programme.

Reach:

- All children in school receiving positive healthy lifestyle messages.
- Parents receiving positive communications about healthy lifestyle messages.

Long term (2+ years)

Fidelity:

- Whole school commitment to delivering healthy eating and physical activities across all key stages using the embedded schemes of work and curriculum activities.
- School committed to working with parents with high quality parent engagement activities planned throughout the year.
- SLT has updated the School Food Policy and is actively monitoring to ensure that School Food Environment meets national nutritional guidelines.
- Phunky AMBASSADORS programme embedded in school.

Final Outcomes (pupils, staff and parents)

Short term (3 months)

- Increased confidence and competence of teaching staff in planning and delivery of healthy lifestyle activities through the active ingredients.

Medium term (6 – 18 months)

- Increased pupil engagement in food literacy curriculum.
- Increased pupil confidence in cooking skills and behaviours.
- Pupils show improved food choices at lunchtimes.
- Improved dietary behaviours of pupils.
- Support staff accredited.
- Parents engaged with healthy lifestyle messages and support children and the school culture and ethos of healthy lifestyles.
- Parents sending healthy lunch boxes to school.

Long term (3-5 years)

- Reduction in Y6 childhood obesity and overweight measured by NCMP.

---

[1] NHS DIGITAL. 2020. *National Child Measurement Programme, England 2019/20 School Year* [Online]. Available: https://digital.nhs.uk/data-and-information/publications/statistical/national-child-measurement-programme/2019-20-school-year [Accessed].

[2] DIMBLEBY, H. 2021. National Food Strategy: The Plan. UK.

[3] SAHOTA, P., CHRISTIAN, M., DAY, R. & COCKS, K. 2019. The feasibility and acceptability of a primary school-based programme targeting diet and physical activity: the PhunkyFoods Programme. *Pilot Feasibility Stud,* 5, 152.

[4] CHARLTON, K., COMERFORD, T., DEAVIN, N. & WALTON, K. 2020. Characteristics of successful primary school-based experiential nutrition programmes: a systematic literature review. *Public Health Nutr*, 1-21.

[5] AXFORD, N., BERRY, V., LLOYD, J., MOORE, D., ROGERS, M., HURST, A., BLOCKLEY, K., DURKIN, AND MINTON, J. 2019. How Can Schools Support Parents' Engagement in their Children's Learning? Evidence from Research and Practice. London: Education Endowment Foundation.
