# Supplementary material for: Evaluation of the PhunkyFoods intervention on food literacy and cooking skills of children aged 7–9 years: a cluster randomised controlled trial in Yorkshire Primary Schools UK
Source: Trials. 2022 Aug 1;23:618. doi: 10.1186/s13063-022-06558-5 (PMC9344772; doi:10.1186/s13063-022-06558-5)
Supplement: Supplementary file 2 — Additional file 2. Consent Materials. [file 13063_2022_6558_MOESM2_ESM.zip › Version 2_HeadTeach_Infor_Sheet_Study 1 KVR2.pdf]

School of Food Science and Nutrition
Faculty of the Environment

UNIVERSITY OF LEEDS

# Cooking in Yorkshire Project	# Headteacher Information

It is important to be aware of information that is provided by the School of Food Science about the general terms and conditions that apply with respect to the processing of personal data. Please consult: https://dataprotection.leeds.ac.uk/

This document explains why we are doing in this research project and sets out what will be involved for the school and the children. We will also provide copies of the information and consent forms that we would like to issue to parents/guardians of the children.

Please note that if, in the course of our study, we discover something that raises concerns about the children's safety or the safety of others, we are obliged to seek further expert help and advice.

1) Background

The University of Leeds would like to invite you to allow certain children at your school to take part in the following research project. The research design is a cluster-Randomised Control Trial. This means that are recruiting 30 schools to the project and the research team will randomly allocate them to group 1 or group 2. Group 1 schools (15) will start the PhunkyFoods Programme in September 2022 with staff training from May 2022. Group 2 schools (15) will start the PhunkyFoods Programme in September 2023 with staff training from May 2023. All schools will participate in data collection in March 2022 and March 2023. Schools will be randomly allocated to either Group 1 or Group 2 in April 2022 by the research team. Before agreeing to take part, please read this information sheet carefully and let us know if anything is unclear or you would like further information.

2) What is the purpose of the study?

We are writing to you from the Nutritional Epidemiology Group, at the University of Leeds.

Childhood obesity rates more than double during primary school. Good nutrition and maintaining a healthy weight in childhood helps to prevent obesity and diet-related ill health later in life (Umer et al., 2017). Knowledge about nutrition and cooking healthy meals helps people to live healthier lives (Hersch et al., 2014; Chaudhary et al., 2020). This research will investigate the impact of the PhunkyFoods programme on diet, nutrition knowledge and cooking skills for primary-aged children.

Parental involvement

We will ask parents complete a short diary of their child's eating for 24 hours to that we can find out about eating habits (taking around ~10 minutes). This will be at two time points: March 2022 and March 2023. We will provide paper copies of the diary for parents and would like you to send these to parents.

School involvement

We would like to ask schools to allocate <u>one whole class</u> for the purpose of research activity – either a year 3 or year 4 class. This will involve participating in the data collection activity at two time points: March 2022 and March 2023. The data collection will take place during a whole class activity, lasting approximately 1 hour on each of the two visits. We will also ask you to send home the 24-hour food diary for parents to complete and return to school.

3) What sort of children are we seeking to involve?

Children in years 3 and 4 who are participating in the PhunkyFoods programme at your school starting in either September 2022 or September 2023.

Cooking in Yorkshire Project	Headteacher Information

4) Who will give consent for a child to take part?

We will obtain consent from the parent or carer and from any child aged 16 years or under via opt out. Parents will be able to opt out of the study by contacting the school via telephone or email. It will be made clear that the study is entirely voluntary and, even having given consent, that any child is free to stop participating at any time without giving a reason. We also need your consent to proceed with the study, and similarly, you can withdraw your school from participation at any time during data collection.

5) What will the children be asked to do?

Children will be asked to complete two surveys:

- Food Literacy Assessment (taking ~30 minutes)
- Cooking skills survey (taking ~15 minutes)

Both of these are validated assessments tools for use with children in primary schools and will be fun activities to complete and relevant for the food literacy curriculum. We hope that these surveys can take place during lessons. This will be at two time points: March 2022 and March 2023. Every effort will be made to ensure that the research sessions are as enjoyable and relaxed as possible for the children. The total testing time should not exceed 50 minutes per child.

6) Who will run the testing sessions?

Either a PhunkyFoods facilitator or Karen Vaughan from the University of Leeds project team will hand these out for children to complete in class.

7) Will you share the child's data with 3rd parties?

No. Data will be accessible to the project team at Leeds only.

8) Will you transfer the data internationally?

No.

9) Will any child be identified in any research outputs?

No.

10) Questions or concerns

If you have any questions about this information sheet or concerns about how children's data is being processed, please contact Dr Charlotte Evans.

Contact Details: Dr Charlotte Evans
School of Food Science,
G11 Stead House, University of Leeds, Leeds LS2 9JT

Phone: 0113 343 3956
E-mail: C.E.L.Evans@leeds.ac.uk
