# Supplementary material for: Evaluation of the PhunkyFoods intervention on food literacy and cooking skills of children aged 7–9 years: a cluster randomised controlled trial in Yorkshire Primary Schools UK
Source: Trials. 2022 Aug 1;23:618. doi: 10.1186/s13063-022-06558-5 (PMC9344772; doi:10.1186/s13063-022-06558-5)
Supplement: Supplementary file 2 — Additional file 2. Consent Materials. [file 13063_2022_6558_MOESM2_ESM.zip › Version 2_Parental_Infor_Sheet_Study 1R2.docx]

| **Cooking in Yorkshire Project** | Parent  Information Sheet |
| --- | --- |
| It is important to be aware of information that is provided by the Department of Food Science about the general terms and conditions that apply with respect to the processing of personal data. Please consult:- [***Privacy Notice for Research***](https://dataprotection.leeds.ac.uk/wp-content/uploads/sites/48/2019/02/Research-Privacy-Notice.pdf) | |
| 1. **Background**   The University of Leeds would like to invite you to allow your child take part in the following research project, which is a Randomised Control Trial design.  Before agreeing to take part, please read this information sheet carefully and let us know if anything is unclear or you would like further information.  Please note that if, in the course of our study, we discover something that raises concerns about your child’s safety or the safety of others, we are obliged to seek further expert help and advice. | |
| 1. **What is the purpose of the study?**   The purpose of the study is to find out how the PhunkyFood programme impacts on dietary habits, nutrition knowledge and cooking skills for children in primary schools. Research shows that good nutrition and maintaining a healthy weight in childhood helps to prevent obesity and diet-related ill health later in life. | |
| 1. **Why is my child being invited to take part?**   Your child has been chosen to participate because we are recruiting Key Stage 2 children in schools that are starting the PhunkyFoods programme either in May 2022 or May 2023. | |
| 1. **Do I have to agree to allow my child to be tested?**   No, participation is optional. If you decide that you would like your child to be part of the study, you do not need to do anything. You can opt out of the study by contacting the school via telephone or email. If you change your mind at any point during the study, you will be able to withdraw your child’s participation without having to provide a reason. | |
| 1. **What will my child be asked to do?**   Your child will be asked to complete two fun surveys about food and cooking twice: once in March 2022 and once in March 2023. | |
| 1. **Where will the research sessions take place?**   Research will take place at school. Every effort will be made to ensure that the research sessions are as enjoyable and relaxed as possible for the children. The surveys are designed for children and the class teacher or researcher will be available to help read out any questions in the survey if needed. It will take around 50 minutes in total. | |
| 1. **Who will run the testing sessions?**   Your child’s class teacher, a member of the Research Team or a PhunkyFoods facilitator will hand out the surveys for children to complete in classroom. | |
| 1. **What will parents be asked to do?**   For all the participating children in the study, we would like to collect information from parents to help us understand more about the sample. We would like to invite you to complete a food diary about the eating habits of your child (taking ~10 minutes in total). We will ask you to complete this food diary twice: once in March 2022 and again in March 2023. | |
| 1. **Will you share my child’s data with 3^rd^ parties?**   No. Data will be accessible to the Research Team at the University of Leeds only.  **Audio recordings**  There will be no audio recordings made. | |
| 1. **Will you transfer my child’s data internationally?**   No. | |
| 1. **Will my child be identified in any research outputs?**   No. | |
| 1. **Questions or concerns**   If you have any questions about this information sheet or concerns about how your child’s data is being processed, please contact Dr Charlotte Evans.  Contact Details: Dr Charlotte Evans  School of Food Science  G11 Stead House, University of Leeds, Leeds LS2 9JT  E-mail: [C.E.L.Evans@leeds.ac.uk](mailto:C.E.L.Evans@leeds.ac.uk) | |
