# Supplementary figures and images for: Evaluation of the PhunkyFoods intervention on food literacy and cooking skills of children aged 7–9 years: a cluster randomised controlled trial in Yorkshire Primary Schools UK
Source: Trials. 2022 Aug 1;23:618. doi: 10.1186/s13063-022-06558-5 (PMC9344772; doi:10.1186/s13063-022-06558-5)

## Slide 1
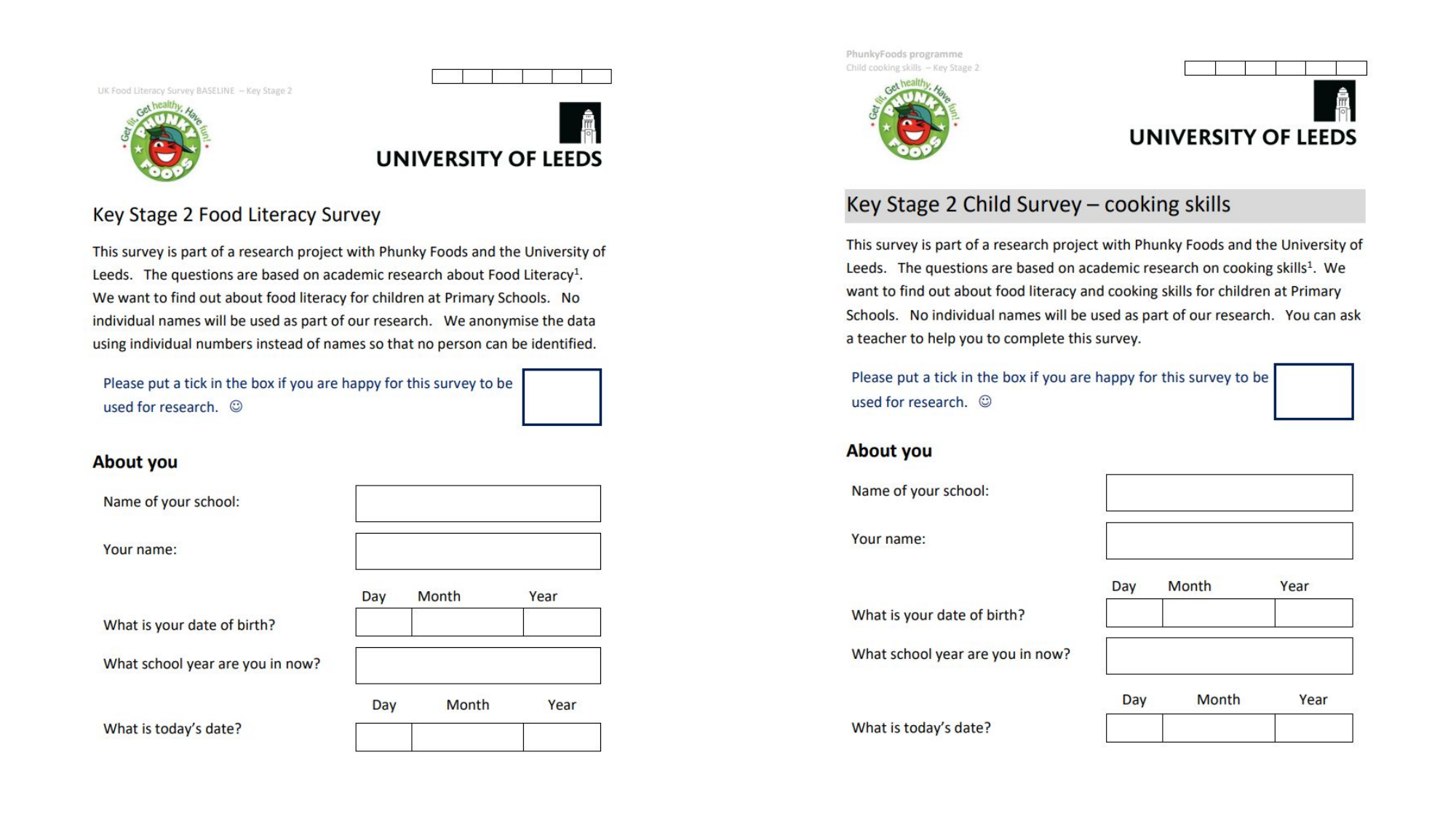

Supplement: Supplementary file 2 — Additional file 2. Consent Materials. [file 13063_2022_6558_MOESM2_ESM.zip › Surveys Child Consent pageR2.pptx]
